# Fabrication of a Cell Fixation Device for Robotic Cell Microinjection

**DOI:** 10.3390/mi7080131

**Published:** 2016-08-04

**Authors:** Yu Xie, Yunlei Zhou, Wenming Xi, Feng Zeng, Songyue Chen

**Affiliations:** School of Aerospace Engineering, Xiamen University, Xiamen 361005, China; 19920141152932@stu.xmu.edu.cn (Y.Z.); wmxi@xmu.edu.cn (W.X.); 19920141152946@stu.xmu.edu.cn (F.Z.); s.chen@xmu.edu.cn (S.C.)

**Keywords:** PDMS, microinjection, robotic, contact force

## Abstract

Automation of cell microinjection greatly reduces operational difficulty, but cell fixation remains a challenge. Here, we describe an innovative device that solves the fixation problem without single-cell operation. The microarray cylinder is designed with a polydimethylsiloxane (PDMS) material surface to control the contact force between cells and the material. Data show that when the injection velocity exceeds 1.5 mm/s, microinjection success rate is over 80%. The maximum value of the adhesion force between the PDMS plate and the cell is 0.0138 N, and the need can be met in practical use of the robotic microinjection.

## 1. Introduction

Microinjection allows the administration of a foreign target gene directly into the nucleus of a fertilized egg using a glass needle under a microscope to integrate the foreign gene into the recipient cell genome. This is a common biological procedure, used in in vitro fertilization and transgenic technology [1]. Historically, the traditional technique involved transferring cells with a microsuction tube under a microscope and microinjecting with a needle smaller than 10 μm in diameter [2]. However, this required specific skills and time and effort. Recently, Sun and Dong’s groups reported successful automation of this technique [3,4,5,6]. Specifically, a motor drives the needle to completely inject the cell, and the cell injection force or microscope vision serves as feedback. Robot-assisted microinjection is an emerging area of research that is capable of improving efficiency, particularly in precision and high-throughput aspects.

Current research into automated cell microinjection has been significant, but challenges remain such as cell fixation during injection. Liu et al. made six symmetrical cylinders to fix the cell, and cell force is calculated according to the displacement of the column during the injection process [7]. Lu et al. adopted a gel cell holder with parallel V-grooves and a machine vision algorithm to identify the number of embryos in a batch and then locate the centerline of each embryo [8]. Xie et al. fabricated continuous and transparent V-shape cell structures on piezoelectric sensors, which can be used for cell injection as well as the detection of injection force in the force control system [9]. Liu et al. used microfluid technology to make some cell holding cavities to patch the cells by the differential pressure between the layer above and below. It is a fabulous solution, but the whole system appears to be quite complicated [10]. In Huang et al., suspended cells were fixed by a specially-designed cell holding device that enabled automatic injection of a batch of suspended cells. To facilitate the pick-and-place process of embryos, holes were embedded in circular profile grooves centered around the geometric center of the holder [11]. In sum, the overall fixing methods are based on negative pressure or the fixation structure. However, most of the methods have a common disadvantage—they all need to move the cell to a fixed position. However, it is quite difficult to transfer a single cell into a tiny cell device by manual operation.

To solve the problem, this paper introduced a device based on the adhesive effect of the cell and material surface to make cells fixed without a single cell transfer. Polydimethylsiloxane PDMS has been widely used in the biomedical field as a transparent and nontoxic material [12,13]. Recent research has shown that the contact force between PDMS and an object can be modified by improving the chemical property and surface pattern [14,15,16]. Wang et al. illustrated the relativity of the friction between the PDMS surface array and a PDMS sphere [17]. This result inspiringly offers us new thought into solving the fixation problem of automatic microinjection. Thus, we fixed a single zebrafish embryo cell using an array. We fashioned microcylinders (1700 × 1700) on a PDMS surface (3.5 cm × 3.5 cm). Cylinders were 10 μm apart. By modifying surface topography, the adhesion force between cells and materials was controlled, and during microinjection, liquid drops containing embryonic cells are placed on the PDMS material using a microsuction tube. In comparison with the traditional fixing methods, a single cell transfer operation is not required, and thus the procedure is simplified to a large extent, which benefits the operator significantly.

## 2. Experimental Methods

### 2.1. Surface Texturing

To make the PDMS surface array cylinder, craters (1700 × 1700) are created as a mold on the surface of a silicon wafer. After spin-coating photoresistance onto the wafer and setting the pattern, a wafer mold is etched using an Inductively Coupled Plasma (ICP) deep etching system (AMS200, Alcatel, Annecy, France). The corrosion gas is SF_6_ (LinDe Gas, Xiamen, China), the protective gas is C_4_H_8_ (LinDe Gas). Two kinds of gases act on the silicon wafer, and the etching and passivation are carried out. The etching depth is 5 μm.

**Figure 1:**
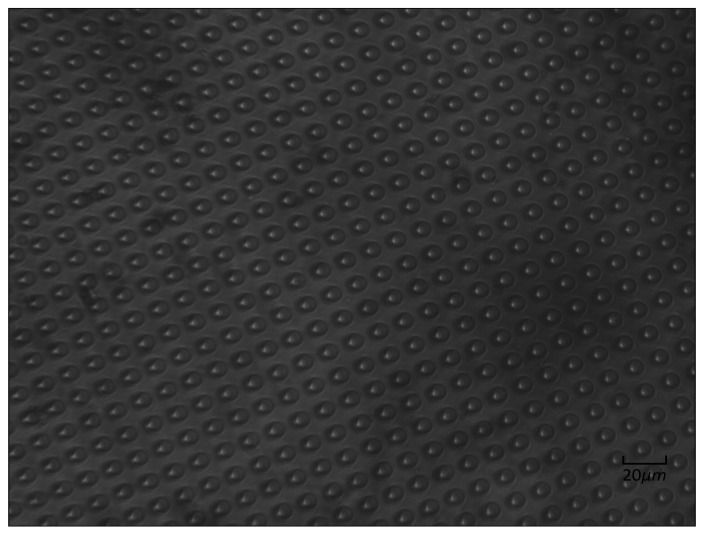
Top-view microscope image of polydimethylsiloxane (PDMS).

PDMS was prepared using a Sylgard 184 (Dow Corning Corp., Midland, MI, USA), and a curing agent was made in a ratio of 12:1 *w/w*. The mixture was placed in a desiccator and degassed for 20 min and then poured into a Petri dish and degassed in a vacuum heating furnace at 70 °C for 12 h. A scalpel was used to evenly cut around the pattern, and the PDMS was removed from the silicon mold. Figure 1 depicts the optical microscope image of PDMS.

### 2.2. Experimental Setup

**Figure 2:**
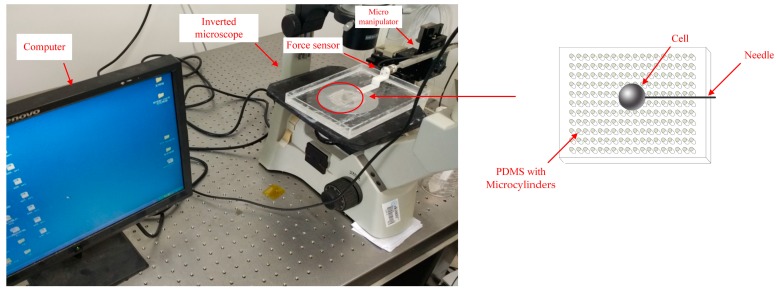
Experimental set-up for zebrafish embryo injection.

**Figure 3:**
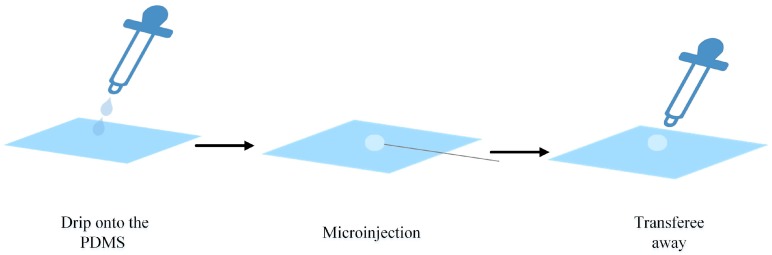
The whole procedure process.

We tested the fixed performance of the PDMS. Zebrafish embryo cells have different features that are elastic and require different puncture forces for each developmental stage. So, the 32-cell-stage was selected for the microinjection. PDMS was placed in a culture dish submerged in deionized water 5 mm below the water surface. This was to exclude the interference of fluid and surface tension. A zebrafish embryonic cell mixture was pulled into a microsuction tube and then dripped on the PDMS. After the cell fell on the PDMS surface, the culture dish was then moved to the microscope (AE31, Motic, Xiamen, China) platform to microinject the targeted embryos. To detect the force of the injection, a force sensor (Nano17, ATI, Apex, NC, USA) was applied between it and the motor. A three-degrees-of-freedom (3-DOF) robotic arm with a 0.04 μm positioning resolution (model: MP-285, Sutter Inc., Novato, CA, USA) drives the injection needle to puncture the cell, and cell injection success or failure was recorded. The experimental injection system appears in Figure 2. The whole procedure is shown in Figure 3; the cell was dripped onto the PDMS surface for the microinjection and transferred away by a suction tube.

The micropipettes are made from a glass tube with an outer diameter of 1.0 mm and an inner diameter of 0.5 mm (TW100F-4, World Precision Instruments, Sarasota, FL, USA), heated and pulled with the use of a micropipette puller (PC-10, Narishige, New York, NY, USA). The micropipette with a tip diameter of 25 μm after grinding is connected to an automatic pressure microinjector (IM-300, Narishige) via a pressure tube.

In the experiment, the cell was injected from the horizontal direction and tested the fixation effect. The angle was chosen because the cell was most prone to move in this condition. Taking the acceleration process into consideration, the injection needle was set 1 mm away from the cell, and the total travel distance during the injection was 1.7 mm.

## 3. Results

**Figure 4:**
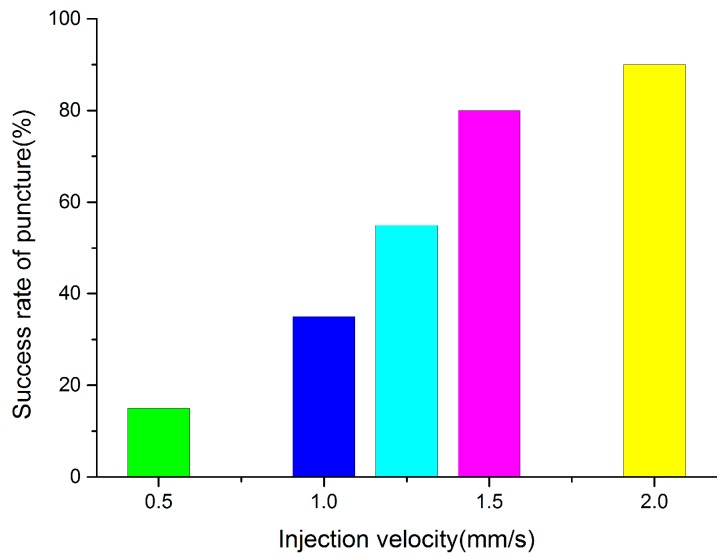
Puncture success rate at different velocities.

Different injection velocities were tested 20 times. Video S1 shows the robot-assisted microinjection experiment with the zebrafish embryo at 1.5 mm/s. From Figure 4, we can find that the success rate of puncture obviously rises as the injection speed increases. This was the result of the increasing acceleration between the embryo and the needle, as well as the interaction force. Injection success was low when the velocity was less than 1 mm/s, and reached 80% at 1.5 mm/s, which meets the application standard of microinjection. A velocity faster than 1.5 mm/s is feasible, so this method has broad application for automated microinjection processes.

**Figure 5:**
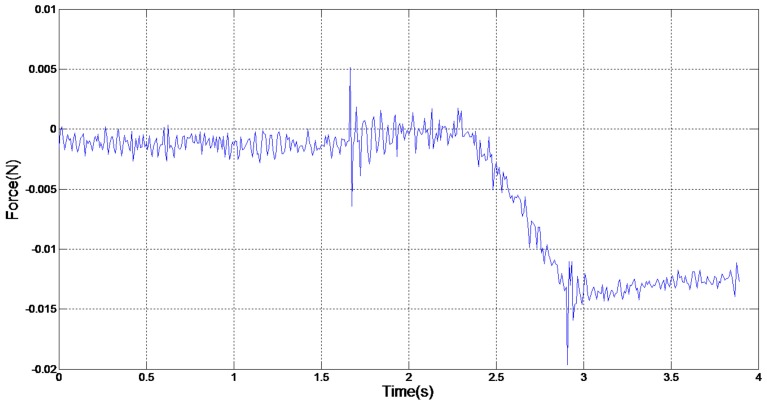
Contact force of failed injection.

A puncture force analysis has been conducted when the velocity is 1.5 mm/s, both in successful and failed cases. Figure 5 shows the case of the contact force between the injection needle and the embryo with a 1.5 mm/s velocity in a failed puncture. The contact force fluctuated when the embryo was exposed to the injection needle and its first movement. This was mainly caused by vibrations of contact and rigid motions. As the injection needle entered the embryo, the embryo membrane was pulled into the embryo, and the contact force between the embryo and the injection needle gradually increased at the same time. When the contact force between the embryo and needle exceeded the contact force between the embryo and the PDMS, the embryo and needle moved together. In this case, the contact force between the embryo and the injection needle was unchanged and both moved together at the same speed.

**Figure 6:**
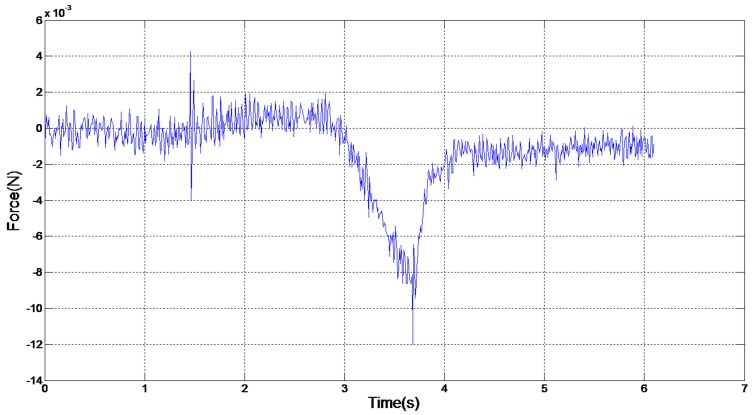
Contact force of successful injection.

Figure 6 shows the classical case of the contact force between the injection needle and the embryo with a 1.5 mm/s velocity in a successful puncture. The contact force gradually increased as the injection needle moved before punctuation and then diminished to indicate successful puncture.

From Figure 5 and Figure 6, the key to a successful puncture lies in the embryo puncture force at a certain velocity. In Figure 4, the injection failed, whereas the injection force reached as large as 0.014 N at 1.5 mm/s. However, the embryo was successfully punctured even when the force was 0.009 N at the same velocity. It is normal for a specific zebrafish embryonic embryo to require a large puncture force to penetrate. It is obvious that the adhesion force between the modified PDMS and embryo is limited, and microinjection fails when the injection force exceeds the maximum value of the adhesion force. From the failed group, we calculate 0.0138 N as the maximum value. The diameter of the injection needle also counts in a successful puncture. Less force is needed in the same embryo when the injection needle is thinner. The diameter in usual biomedical experiments is 20 μm, and a size of 25μm is used in this paper; so, the basic standard can be met in practical use. In terms of puncture force, the device we fabricated was again proven to be feasible in robotic microinjection.

## 4. Conclusions

We fabricated an array cylinder on the light transmitting PDMS material to regulate the force between the material and fixed embryos during automatic zebrafish embryo microinjection. The success rate was 80% when the injection velocity was 1.5 mm/s. The maximum value between the PDMS surface and the cell is 0.0138 N, which meets the requirements in the practical use of robotic microinjection.

## Supplementary Materials

The following are available online at http://www.mdpi.com/2072-666X/7/8/131/s1, Video S1: Successful injection at 1.5 mm/s.
